# A Novel Approach to Study Coherent γ-Band Oscillations in Hippocampal Brain Sections

**DOI:** 10.1523/ENEURO.0167-23.2023

**Published:** 2023-07-21

**Authors:** Jean C. Rodríguez Díaz, Paul M. Jenkins, Dominique L. Pritchett, Kevin S. Jones

**Affiliations:** 1 Neuroscience Graduate Program, University of Michigan Medical School, Ann Arbor, 48109 MI; 2 Department of Pharmacology, University of Michigan Medical School, Ann Arbor, 48109 MI; 3 Department of Psychiatry, University of Michigan Medical School, Ann Arbor, 48109 MI; 4 Biology Department, Howard University, Washington, DC 20059

**Keywords:** ankyrin, bipolar disorder, electrophysiology, γ oscillations, multielectrode array, schizophrenia

## Abstract

γ-Band oscillations (GBOs) are generated by fast-spiking interneurons (FSIs) and are critical for cognitive functions. Abnormalities in GBOs are frequently observed in schizophrenia and bipolar disorder and are strongly correlated with cognitive impairment. However, the underlying mechanisms are poorly understood. Studying GBOs in *ex vivo* preparations is challenging because of high energy demands and the need for continuous oxygen delivery to the tissue. As a result, GBOs are typically studied in brain tissue from very young animals or in experimental setups that maximize oxygen supply but compromise spatial resolution. Thus, there is a limited understanding of how GBOs interact within and between different brain structures and in brain tissue from mature animals. To address these limitations, we have developed a novel approach for studying GBOs in *ex vivo* hippocampal slices from mature animals, using 60-channel, perforated microelectrode arrays (pMEAs). pMEAs enhance oxygen delivery and increase spatial resolution in electrophysiological recordings, enabling comprehensive analyses of GBO synchronization within discrete brain structures. We found that transecting the Schaffer collaterals, a neural pathway within the hippocampus, impairs GBO coherence between CA1 and CA3 subfields. Furthermore, we validated our approach by studying GBO coherence in an *Ank3* mutant mouse model exhibiting inhibitory synaptic dysfunction. We discovered that GBO coherence remains intact in the CA3 subfield of these mutant mice but is impaired within and between the CA1 subfield. Overall, our approach offers significant potential to characterize GBOs in *ex vivo* brain sections of animal models, enhancing our understanding of network dysfunction in psychiatric disorders.

## Significance Statement

Synchronized brain activity is crucial for various cognitive behaviors, and abnormalities in γ-band oscillations (GBOs) are prevalent in numerous mental health disorders. Our study presents an innovative method that utilizes microelectrode arrays (MEAs) to record GBOs across multiple locations within the hippocampus. This approach allows us to investigate the development of GBO coherence within and between specific subregions of the hippocampus, providing a more comprehensive understanding of how brain activity is synchronized in both healthy rodents and animal models of neurologic and psychiatric diseases.

## Introduction

The brain’s neurons form intricate networks that generate and coordinate electrical signals, exhibiting various oscillatory patterns categorized into distinct frequency bands, including θ (4–8 Hz), α (8–12 Hz), β (14–30 Hz), and γ (30–100 Hz; Buzsáki, 2006; Jensen et al., 2019). Of particular importance are γ-band oscillations (GBOs), which correlate with and play a critical role in cognitive behaviors such as attention, memory, and sensory processing (Tallon-Baudry et al., 1998; Cardin et al., 2009; Fries, 2009; Buzsáki and Wang, 2012; Siegle et al., 2014; Pritchett et al., 2015). Notably, psychiatric disorders frequently exhibit abnormal GBOs, suggesting their involvement in the pathophysiology of conditions such as schizophrenia (Spencer et al., 2009; Spencer, 2011; Grützner et al., 2013), bipolar disorder (Liu et al., 2012; Nelson et al., 2020), and Alzheimer’s disease (Goutagny et al., 2013; Klein et al., 2016).

GBOs can be investigated in *ex vivo* hippocampal sections and retain many characteristics of their *in vivo* counterparts (C.B. Lu et al., 2011). However, generating GBOs in *ex vivo* preparations is challenging because of the substantial increase in metabolic load and oxygen consumption during GBO generation (Kann et al., 2011), and sufficient oxygen must be delivered to meet these heightened energy demands (Hájos et al., 2009; Kann et al., 2016). Interface chambers have been used to overcome this obstacle by directly exposing the *ex vivo* tissue to highly oxygenated air (Buhl et al., 1998; Fisahn et al., 1998). Extensive research using interface chambers has explored GBOs in *ex vivo* hippocampal preparations (Buhl et al., 1998; Fisahn, 2005; Tsintsadze et al., 2015). However, solution exchange is slow in interface chambers, often requiring 1–2 h of drug delivery to pharmacologically induce GBOs (C. Lu et al., 2012; Pietersen et al., 2014; Lemercier et al., 2017; Xie et al., 2021). Submerged hippocampal sections allow faster pharmacological induction of GBOs (C. Lu et al., 2012; Cabungcal et al., 2013), but the limited availability of oxygen within the tissue cannot sustain continuous GBOs. Consequently, submerged preparations are primarily used to study transient GBOs triggered by brief electrical stimulation (Carmeli et al., 2013) or localized application of agonists (Gloveli et al., 2005; McNally et al., 2011).

To gain a better understanding of the generation and propagation of pharmacologically-induced GBOs throughout the complex structure of the hippocampus, we developed an approach with enhanced spatial resolution. Our approach combines the use of perforated microelectrode arrays (pMEAs) and fast superfusion, enabling the delivery of oxygenated artificial CSF (aCSF) and pharmacological agents through the interstitial space of brain sections. The electrode geometry on the MEA chips allowed for simultaneous recordings from multiple locations within the cornu ammonis (CA) CA1 and CA3 subfields of the hippocampus, which significantly improves spatial resolution. This enabled us to investigate the dynamic coupling of GBOs and confirm that GBO coherence between the CA1 and CA3 subfields, but not within each subfield, is modulated by severing the Schaffer collaterals. To the best of our knowledge, this report is the first to examine the temporal dynamics of GBO coherence within and between hippocampal subfields in *ex vivo* brain preparations from adult mice. Furthermore, we demonstrate the translational potential of our approach by characterizing abnormalities in GBOs evoked in hippocampal sections from a mouse model with *Ank3*-dependent inhibitory synaptic dysfunction.

## Materials and Methods

Mice were housed in the University of Michigan’s animal care facilities with controlled temperature and lighting conditions (12/12 h light/dark cycle). Mice had access to food and water *ad libitum*. All animal procedures were conducted in accordance with the guidelines of the University of Michigan’s Institutional Animal Care and Use Committees (IACUC) and complied with NIH *Guidelines for Animal Use*. The mice used in the study were 3-7 weeks old. C57BL/6J mice (IMSR catalog #JAX:000664, RRID: IMSR_JAX:000664) were used for assay development. *Ank3* p.W1989R knock in mice were used to model the impact of inhibitory synaptic dysfunction on GBO function.

### Electrophysiological recordings

Electrophysiological recordings were captured on pMEAs comprised of 59 titanium nitride recording electrodes (30 μm in diameter) and one reference electrode arranged in a 6 × 10 grid with a 100-μm interelectrode distance (Multichannel Systems Reutlingen, Germany). One electrode serves as an internal reference. Male mice were anesthetized with isofluorane and intracardially perfused with ice-cold (4°C) modified N-Methyl-D-glucamine (NMDG) HEPES artificial CSF (aCSF) consisting of in mm: 93 NMDG, 2.5 KCl, 0.5 CaCl_2_, 10 MgCl_2_, 1.2 H_2_PO_4_, 20 HEPES, 25 dextrose, 5 ascorbic acid, 2 thiourea, and 3 Na-pyruvate (Ting et al., 2018). pH was maintained at 7.4 by saturation with O_2_/CO_2_ (95/5%, respectively). Mice were quickly decapitated, and the brain was dissected and transferred to a holding chamber with ice-cold NMDG HEPES aCSF. Horizontal hippocampal sections (300 μm thick) were prepared with a vibrating microtome (Leica VT1200). We bi-laterally hemisected brain sections and transferred them to an intermediate holding chamber filled with NMDG HEPES aCSF maintained at 33°C for 10–12 min. For experiments where the Schaffer collaterals were transected, we used a sterile scalpel to make a 3- to 5-mm incision along the dorsoventral axis beginning at the pial surface near CA1. Sections were transferred to an intermediate holding chamber filled with aCSF consisting of in mm: 126 NaCl, 3 KCl, 2 CaCl_2_, 1 MgCl_2_, 1.25 NaH_2_PO_4_, 25 NaHCO_3_, and 10 dextrose at 33°C for 35 min. Sections were transferred to ambient temperature for at least 15 min before recording. We mounted sections on pMEAs (Multichannel Systems) and secured them to the surface using a peristaltic perfusion system (PPS2, Multichannel Systems) to create a slight vacuum through the perforations. Sections were superfused in aCSF at a rate of 5–7 ml/min (29–31°C). Recordings were acquired at 20 kHz with a MEA2100 head stage (Multichannel Systems), and the digitized signals were recorded on the hard disk of a personal computer for offline analysis. Baseline recordings were obtained for 1 h. Chemically-induced oscillations were evoked by bath application of kainate (400 nm) for 1 h. All solutions were bubbled with with 95%O2/5% CO_2_ to maintain pH 7.4.

### Data processing

Oscillations from ∼25–150 Hz have been designated for the γ band, and the “slow” γ band comprises frequencies of ∼25–60 Hz (Colgin et al., 2009). We focused our analysis on frequencies from 25–59 HZ, as oscillations in this range, can be elicited by kainate in *ex vivo* hippocampal sections (McNally et al., 2011), and to omit 60 Hz line noise. Power analysis was done by multitaper spectrum analysis using custom-written MATLAB scripts and the multi-tapered Fourier estimation (http://chronux.org/; Mitra and Bokil, 2008). The absolute power in the γ band was calculated from the integral of the power spectrum between 25 and 59 Hz. Data were preprocessed on a workstation and analyzed using MATLAB (MathWorks) and exported to GraphPad Prism for statistical analysis and plotting, unless otherwise specified. Local field potentials were isolated from MEA recordings by applying a low pass IIR Butterworth filter at 100 Hz and down-sampled to 1 kHz on each channel. Spectrograms were made by convolving the signals with a morelet wavelet as follows: $w\left(t,f_{0}\right)=A\exp\left(-t^{2}/2\sigma_{t}^{2}\right)\text{exp}(2i\pi f_{0}t)$, where A is a normalization factor equal to $(\sigma_{t}\sqrt{\pi }){-}^{\frac{1}{2}}$. The width of the wavelet was set to 25, $m=f_{0}/\sigma_{f}$ with $\sigma_{f}=\frac{1}{2}\pi \sigma_{t}$. Interhemispheric, zero-phase-lag coherence of γ oscillations was computed between the LFP on all electrodes on the MEA using the multitapered approach above (http://chronux.org/; Mitra and Bokil, 2008).

We used the final 5 min of drug application to calculate the fractional difference in oscillatory power using the equation $Power_{Drug}-Power_{Drug}/Power_{Vehicle}$, where the drug was kainate or kainate and bicuculline. GBO coherence was obtained from data acquired during the last 5 min of kainate application. We used the Chronux toolbox and calculated the autocorrelation on each channel, Sx(f) and Sy(f), and the cross-spectrum between the channels Sxy(f), which is calculated as follows: $Cxy\left(f\right)=Sxy(f)/\sqrt{Sx\left(f\right)Sy\left(f\right)}$ (Gregoriou et al., 2009). Calculations for the GBO power coherence were done on the Great Lakes High Performance Computing Cluster at the University of Michigan.

We characterized GBO onset by evaluating GBO_90_—the time required for GBO power to attain 90% of the maximum value. GBO_90_ was obtained by fitting the power in the γ band to the sigmoid function $\frac{c}{1+\text{exp}(-a*x+a*b)}+d$. Recordings from channels exhibiting transient excessive noise in the γ band were cleaned using linear interpolation for the missing data points, or discarded if the sigmoidal fit could not be performed. The fit window and the starting parameters for estimating the sigmoid fit were obtained manually for each recording. GBO_90–10_ was obtained from the falltime function in MATLAB and used to measure the interval between GBO power rising and falling above thresholds of 90% and 10% of maximum, respectively. GBO_90–10_ and GBO_10_ were derived from data acquired during the last 2 min of bath application of kainate and the final 10-min application of kainate + bicuculline. The Q factor of the oscillations (Johnson, 1947; Kneubühl, 1997; Shea, 1929) was calculated by the equation Q = f0/B, where f0 is the peak frequency, and B is the bandwidth at 50% of maximum peak power. Recordings obtained from electrodes with artifacts or excessive noise in the γ band were excluded from the kinetic analysis.

### Statistical tests and models

Wilcoxon signed-rank test was used for paired data. Kolmogorov–Smirnov or Kruskal–Wallis tests were used for unpaired data, followed by Dunn’s multiple comparisons for grouped data. Linear mixed-effects models were used to calculate GBO coherence within and between CA1 and CA3 with the following datasets: assay development, transected Schaffer collaterals, and *Ank3* mutants. All linear mixed-effects models were generated using MATLAB’s fitlme function, and maximum likelihood estimation was used as the fitting method. The full covariance matrix was calculated using the Cholesky parameterization. Results of *t* tests (testing the null hypothesis that the coefficient is equal to zero) are reported in Tables 1-Tables 3, adjacent to fixed and random effect coefficients. All three models incorporated the same random variables: tissue section (slice), the first electrode in the electrode pair used for coherence calculation nested in the slice variable. The linear mixed effect model results are reported in Tables 1-Tables 3. Predictor variables in each model differed as follows: The first model used the region pair coherence calculation (within CA1, within CA3, or between CA1 and CA3) as the predictor variables. The second and third models incorporated a second set of predictor variables and incorporated interactions between the two predictor variables. In the second model, the additional predictor variable was Schaffer collaterals (transected vs intact). In the third model, the additional predictor variable was mouse genotype [wild-type (WT) vs mutant]. Linear mixed-effects models were performed in MATLAB. Custom MATLAB scripts are available at https://github.com/Jcrd25/NeuroMEACode.

**Table 1:** Linear mixed effect model of GBO coherence within and between hippocampal subfields

Linear mixed-effects model fit by ML							
Model information:							
Number of observations	3349						
Fixed effects coefficients	3						
Random effects coefficients	248						
Covariance parameters	3						
Formula:							
Coherence ∼ 1 + Regions + (1 | Slice) + (1 | Slice:Elec1)							

Model fit statistics:							
AIC	BIC	LogLikelihood	Deviance				
−1644.6	−1607.9	828.31	−1656.6				

Fixed effects coefficients (95% CIs):							
Name	Estimate	SE	*t* stat	df	*p* value	Lower	Upper
‘(Intercept)’	1.231	0.054	22.8	3346	<0.001	1.125	1.337
‘Regions_CA1’	0.031	0.025	1.2	3346	0.225	−0.019	0.080
‘Regions_CA1_3’	−0.104	0.007	−14.2	3346	<0.001	−0.119	−0.090

Random effects covariance parameters (95% CIs):							
Group: Slice (11 Levels)							
Name1	Name2	Type	Estimate	Lower	Upper		
‘(Intercept)’	‘(Intercept)’	‘std’	0.171	0.111	0.264		

Group: Slice:Elec1 (237 Levels)							
Name1	Name2	Type	Estimate	Lower	Upper		
‘(Intercept)’	‘(Intercept)’	‘std’	0.141	0.127	0.158		

Group: Error							
Name	Estimate	Lower	Upper				
‘Res Std’	0.175	0.170	0.179				

## Results

### Facilitating perfusate delivery with perforated MEAs

**Figure 1.**
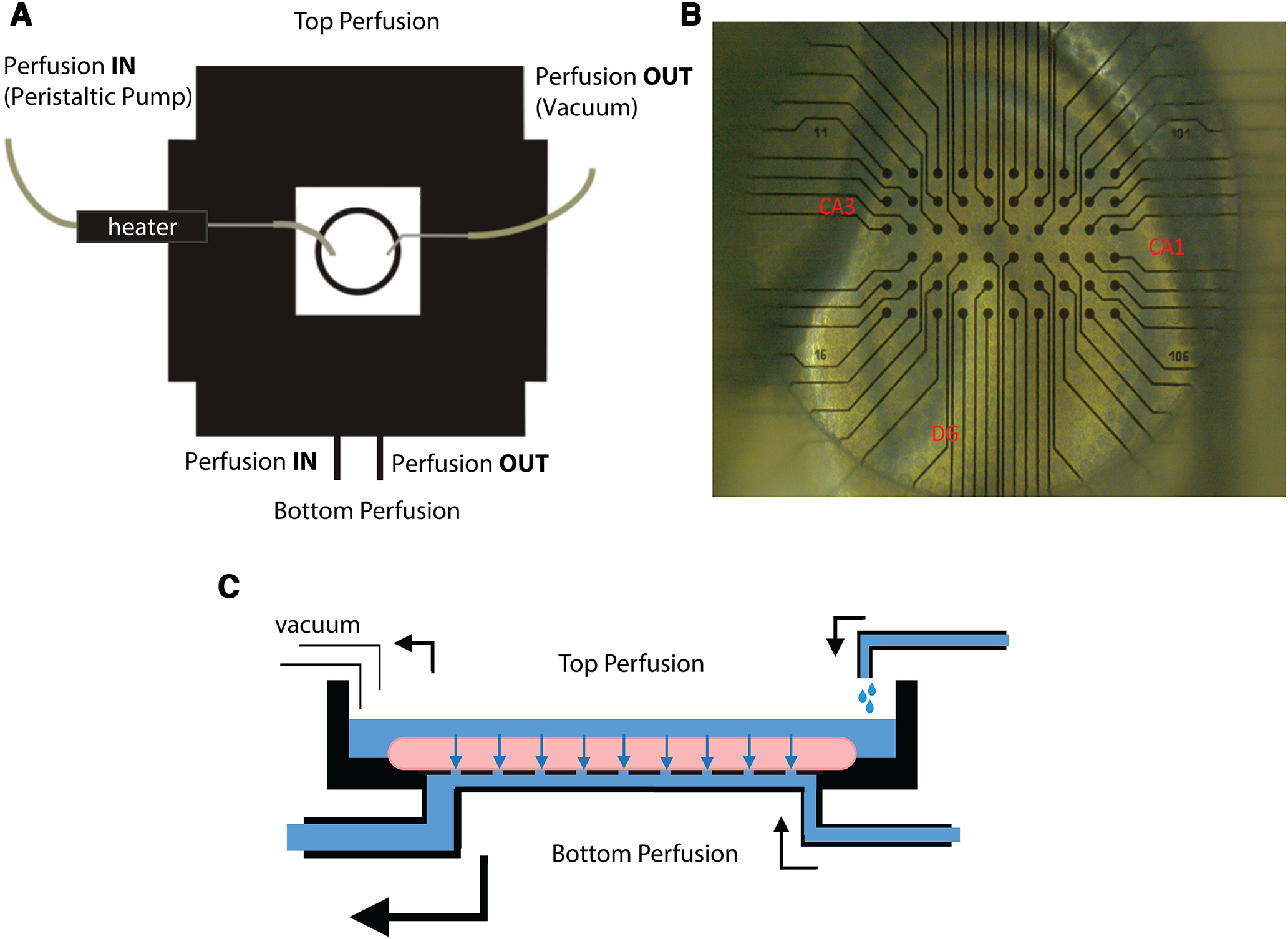
Schematic of experimental apparatus. ***A***, Perfusate is delivered to the MEA chamber via two independent routes. Warm perfusate is delivered to the top chamber of the pMEA through an inline heating element. Perfusate is delivered to the bottom chamber of the MEA in an independently-controlled, semi-closed loop. ***B***, Prototypical layout of the pMEA with a hippocampal slice. Sections were arranged on the electrode array to maximize electrode placement in CA1 and CA3 subfields. ***C***, Schematic of the pMEA chip. Independently-controlled perfusion systems deliver aCSF above and below the MEA chamber. Perfusate is removed from the bottom chamber at a faster rate than it is delivered. The difference in rate creates a slight vacuum in the bottom chamber, drawing perfusate from the top chamber through the tissue, the perforated membrane, and into the bottom chamber. This results in increased diffusion of oxygenated perfusate throughout the tissue.

The pMEA chips used in our study consist of upper and lower perfusion chambers separated by a thin, perforated membrane. We employed a dual-perfusion system to deliver oxygenated artificial CSF (aCSF) both above and below the tissue sections (Fig. 1*A*). The top chamber received warm aCSF, while an independent perfusion system controlled the delivery and removal of aCSF from the bottom chamber. The electrodes on the pMEA were arranged in a 6 × 10 rectangular grid, allowing us to position ∼25 electrodes in the CA3 and CA1 hippocampal subfields (Fig. 1*B*). By removing perfusate from the bottom chamber more rapidly than it was delivered, a slight vacuum was created, which drew aCSF from the top chamber through the brain section, securing it to the array (Fig. 1*C*).

### Induction of GBOs through kainate bath application

**Figure 2.**
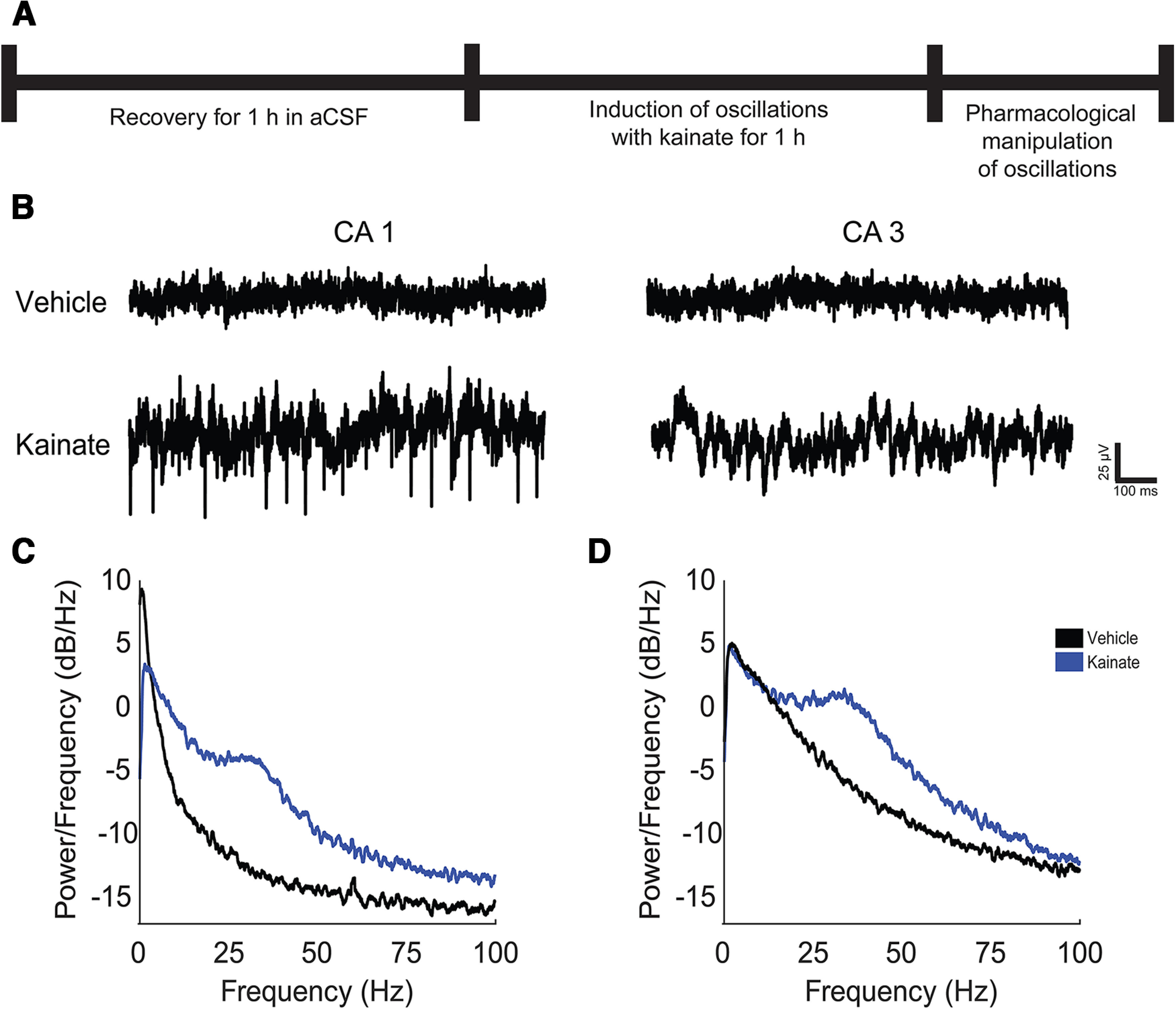
Kainate evokes oscillations in CA1 and CA3. ***A***, Experimental timeline consisted of 1-h acclimation period followed by the perfusion of 400 nm kainate to evoke oscillations for 1 h followed by a 12- to 15-min period of pharmacological manipulation. ***B***, Prototypical extracellular recordings during vehicle or kainate in CA1 and CA3 (low-pass filtered, 100 Hz). ***C***, ***D***, Prototypical periodograms generated from recordings acquired during bath application of vehicle (black) or kainate (blue) in (***C***) CA1 and (***D***) CA3.

**Figure 3.**
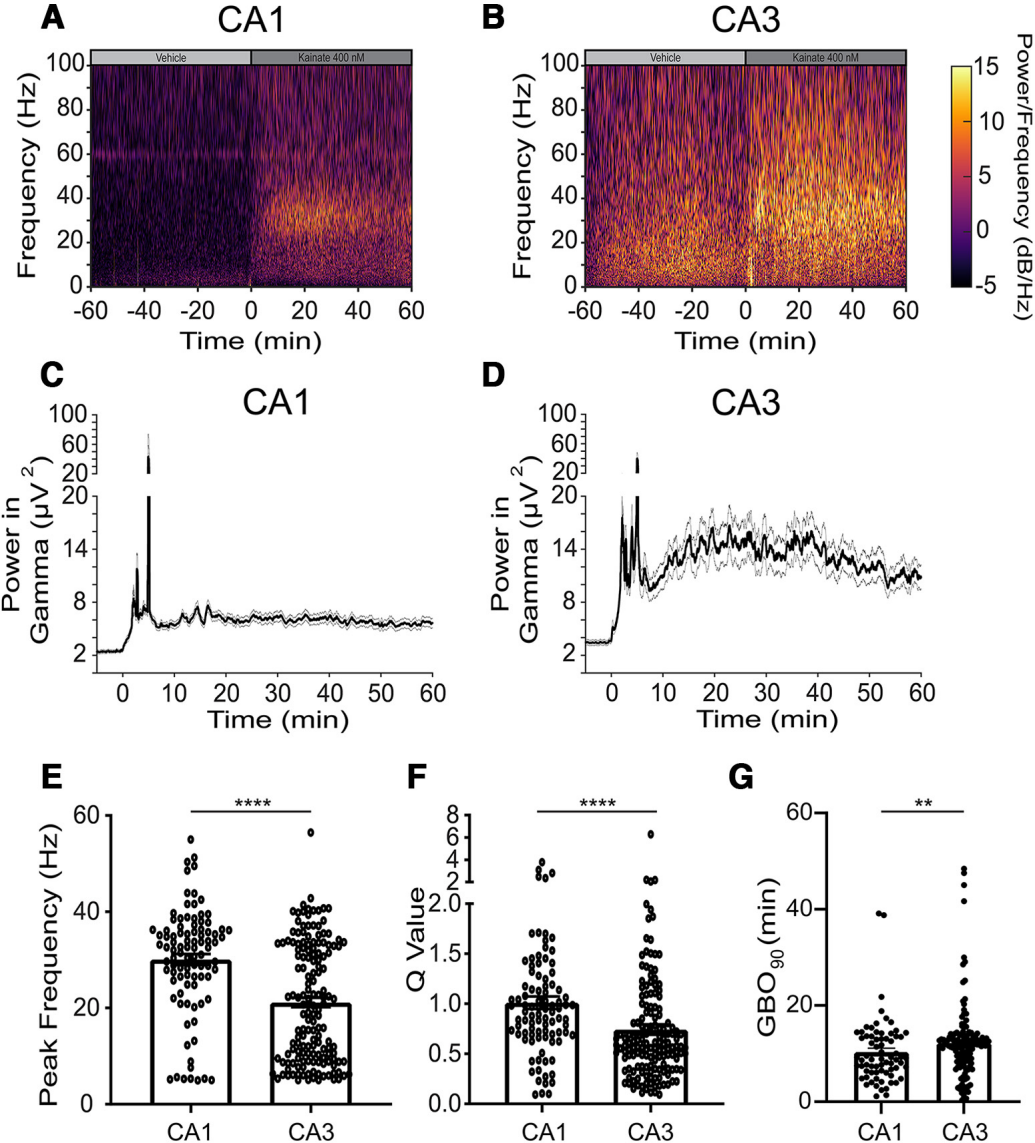
Kainate evokes GBOs in CA1 and CA3. ***A***, ***B***, Prototypical spectrogram from a single electrode in CA1 (***A***) or CA3 (***B***) during bath application of vehicle or kainate (400 nm). ***C***, ***D***, GBO power from (***C***) CA1 (*N* =10, *n *=* *93) and (***D***) CA3 (*N *=* *10, *n *=* *155). ***E–G***, Quantitative characterization of kainate-evoked, γ-band oscillations. ***E***, GBO peak frequency CA1: 30.1 ± 1.15 Hz (*N *=* *10, *n *=* *93 electrodes); CA3: 21.1 ± 1.0 Hz (*N *=* *10 slices, *n *=* *155 electrodes; *p *<* *0.0001). ***F***, Q value CA1: 1.01 ± 0.06 (*N* = 10, *n* = 93); CA3: 0.74 ± 0.05 (*N *=* *10 slices, *n* =155 electrodes; *p* < 0.0001). ***G***, GBO_90_: CA1 10.3 min ± 0.79 *N = *9 slices*, n = *71 electrodes; CA3: 12.2 min ± 0.67 *N = *9 slices*, n = *138 electrodes; *p *=* *0.0073. Kolmogorov–Smirnov test was used to evaluate statistical significance; ****p < 0.0001, **p <* *0.001.

LFPs recorded using planar MEAs typically exhibit smaller amplitudes compared with those obtained with aCSF-filled glass micropipettes. In mouse hippocampal sections, micropipette-recorded GBOs can range from ∼100 μV (Zemankovics et al., 2013) to over 4 mV in optogenetically-stimulated signals (Betterton et al., 2017). In contrast, LFPs captured by planar MEAs have amplitudes ranging from 25–50 μV in both hippocampal (Rolston et al., 2009) and cortical sections (Carmeli et al., 2013). *Ex vivo* hippocampal sections generally exhibit low levels of basal electrical activity (Herman and van Amsterdam, 2014). During vehicle application, we observed minimal spontaneous oscillatory activity across most frequencies (Fig. 2). However, on the application of kainate to the bath, we noticed a pronounced increase in broadband LFP power in the CA1 and CA3 subfields of the majority of sections (10 out of 11). This heightened activity stabilized into consistent oscillatory patterns (Figs. 2, 3*A*,*B*). By visually inspecting the periodograms derived from these recordings, we observed a significant enhancement in LFP power within the frequency range of 20–60 Hz, indicating the successful generation of GBOs in both the CA1 and CA3 subfields (Fig. 2*C*,*D*).

### Continuous GBOs elicited by kainate in adolescent mouse hippocampal sections

To visualize and compare the kinetics of kainate-induced oscillations in the CA1 and CA3 subfields, we generated spectrograms of the LFP recordings (Fig. 3*A–D*). The spectrograms revealed that kainate administration triggered persistent GBOs in both CA1 and CA3 throughout the duration of the experiment. Notably, stable GBOs emerged in CA1 earlier than in CA3 (Fig. 3*G*), and the peak frequency of GBOs in CA1 was ∼50% higher (Fig. 3*E*). We assessed the periodicity of these oscillations using the quality factor, also known as the “Q factor,” a parameter that quantifies the ratio of the peak frequency to the half bandwidth and indicates periodicity in oscillators (Kneubühl, 1997). Previous findings demonstrated that bath application of kainate-induced oscillations in rat hippocampal LFP signals with Q factors >0.5 (Lemercier et al., 2017). We calculated the Q factor values from the last 10 min of kainate bath application using the LFP recordings. In CA1 and CA3, the LFPs exhibited Q factor values of 1.01 ± 0.06 and 0.74 ± 0.05, respectively (*p *<* *0.0001; Fig. 3*F*). These results clearly indicate that bath-applied kainate can generate continuous GBOs in the hippocampus.

### Rapid modulation of kainate-evoked GBOs through drug application

**Figure 4.**
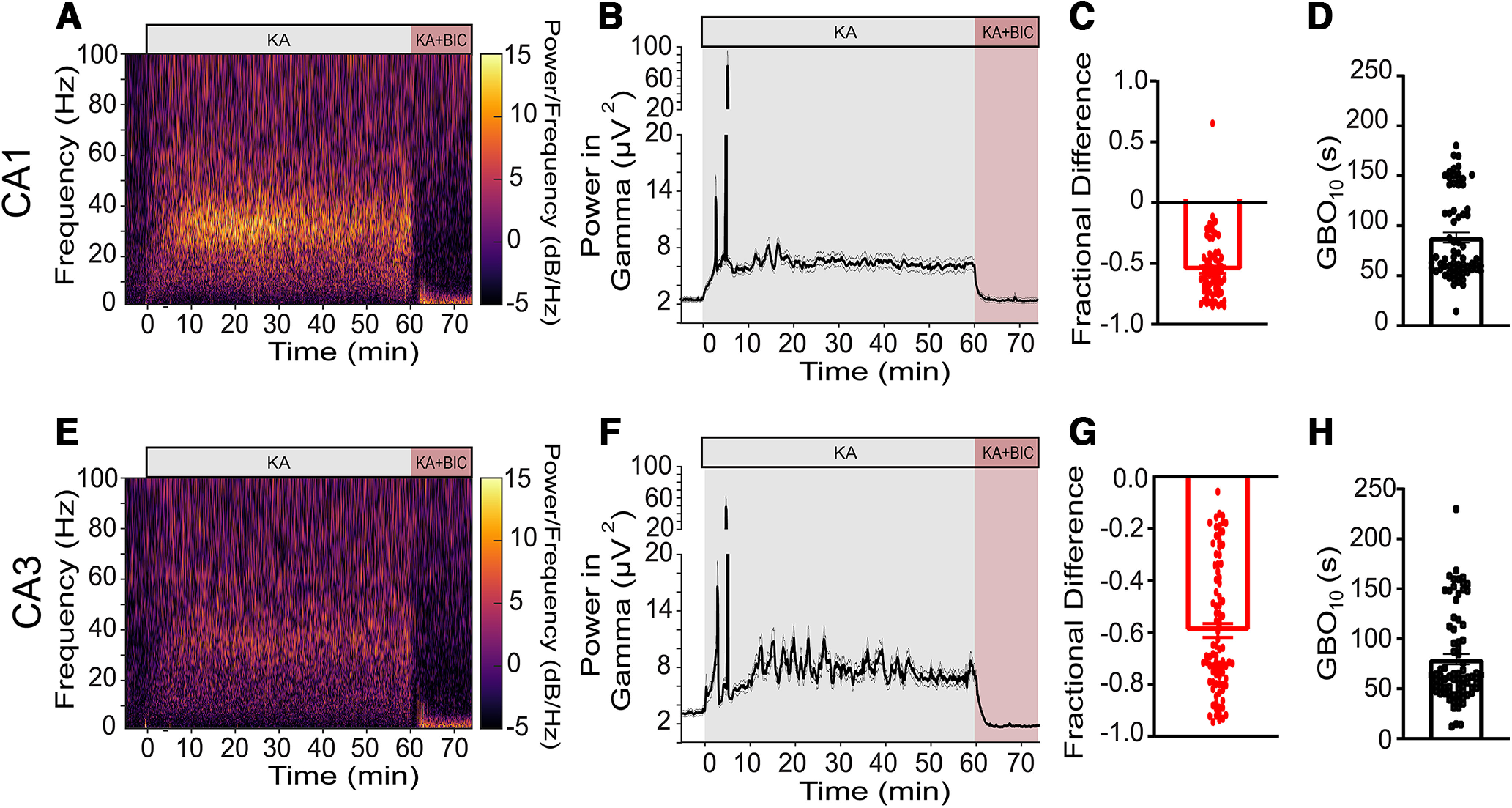
Kainate-evoked GBOs can be rapidly modulated by bicuculline. ***A***, ***E***, Prototypical spectrogram of an electrode in CA1 (***A***) or CA3 (***E***). ***B***, ***F***, Mean kinetics of normalized GBO power in kainate (400 nM) or kainate + bicuculline (10 μM) in CA1 (***B***; *N *=* *7, *n* = 71) and CA3 (***F***) *N *=* *7, *n *=* *88). Bicuculline attenuates hippocampal GBOs (***C, G***). Bicuculline attenuates GBO power in CA1 (***C***) −0.55-fold (± 0.03) (*N *=* *7, *n* = 71), and in CA3 (***D***) −0.59-fold (± 0.02) (*N* = 7 slices, *n* = 88 electrodes). The time course of the pharmacological action of bicuculline (GBO_10_) in CA1 (***D***) was 62.91 ± 3.1 s (*N* = 7, *n* = 69), and in CA3 (***H***) was 79.6 ± 5.2 s, (*N* = 7 slices, *n* = 77 electrodes). Data reported as mean ± sem.

One notable advantage of using *ex vivo* tissue preparations is the ability to swiftly administer drugs for the pharmacological modulation of GBOs. In our experimental setup, we investigated the speed at which kainate-induced GBOs could be modulated by co-applying the GABA_A_ receptor antagonist, bicuculline, to the perfusate (Fig. 4). Bath application of bicuculline attenuated ∼60% in GBO power generated in both CA1 and CA3 (Fig. 4*C*,*H*). By analyzing the kinetics of bicuculline action, we determined that 90% of its pharmacological effect was achieved in <90 s (Fig. 4*D*–*H*). These findings highlight the remarkable speed at which water-soluble compounds can be delivered within our experimental setup and further support the critical involvement of GABA_A_ receptors in hippocampal GBOs (Buhl et al., 1998; Fisahn et al., 1998; C. Lu et al., 2012). Notably, Hájos and colleagues previously described a custom-made perfusion chamber that expedites the onset of pharmacologically-evoked GBOs (Hájos and Paulsen, 2009).

### Kainate induces synchronization of GBOs within and between hippocampal subfields

**Figure 5.**
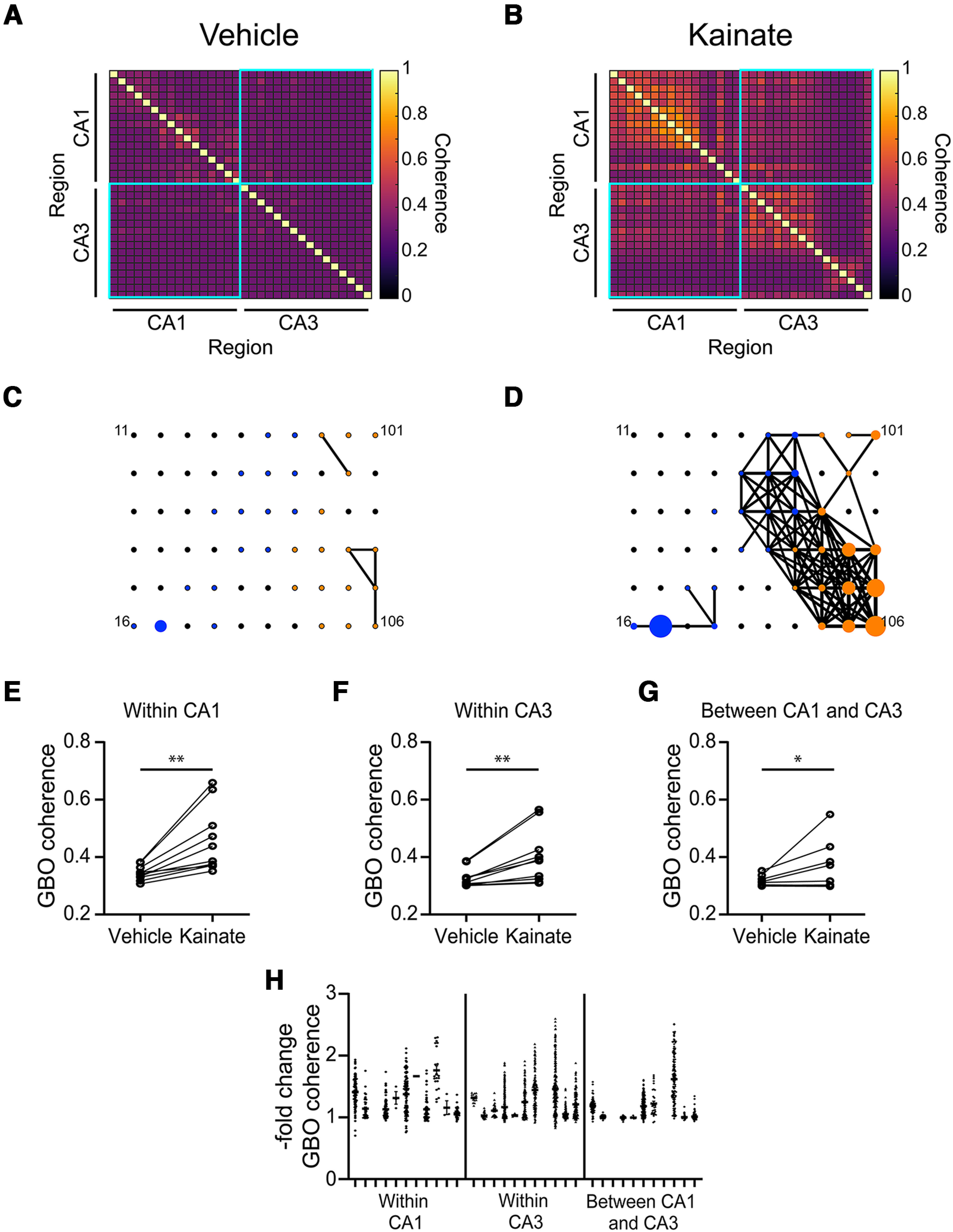
Kainate increases coherence of GBO power between CA1 and CA3. ***A***, ***B***, Representative correlations of GBO power between MEA electrodes in vehicle (***A***) or kainate (***B***). ***C***, ***D***, Representative graphical depiction of GBO power coherence within and between CA1 and CA3 in vehicle (***C***) or kainate (***D***). Electrodes in CA3 are represented by blue circles. Electrodes in CA1 are represented by orange circles. Symbol size is proportional to GBO power. Lines connect electrodes with power coherence ≥0.45. ***E–G***, Kainate-evoked changes in mean GBO coherence of electrodes located within CA1 (***E***; GBO coherence vehicle: 0.345 ± 0.008; kainate: 0.457 ± 0.036; *n* = 10; *p *=* *0.002); within CA3 (***F***; vehicle: 0.328 ± 0.010; kainate: 0.401 ± 0.030; *n* = 10; *p *=* *0.0020); and between CA1 and CA3 (***G***; vehicle: 0.316 ± 0.006; kainate: 0.362 ± 0.028; *n* = 9; *p *=* *0.027). Data are mean ± SEM, *n* = number of slices. Wilcoxon test. ***H***, Fold change in GBO coherence for each pair-wise comparison of electrodes by location within hippocampal subfields. Individual circles represent pair-wise correlation of GBO between two electrodes. See Table 1 for results from linear mixed effects model.

GBOs can transiently emerge within local networks of neurons, but they can also interact and synchronize across distinct networks (Buzsáki and Wang, 2012). Leveraging the geometric spacing between the electrodes on our MEA chips, we simultaneously recorded signals from multiple locations within the CA1 and CA3 hippocampal subfields. By analyzing LFP signals from channels in CA1 and CA3, we characterized how kainate-induced GBOs synchronize within and between these hippocampal subfields. During basal conditions, the coherence of GBO power was generally low within and between CA1 and CA3 (Fig. 5*A*,*C*). However, on kainate application, we observed a rapid increase in GBO power coherence (Fig. 5*B*,*D*–*F*). Specifically, kainate enhanced the mean GBO power coherence within CA1 by ∼32% and within CA3 by ∼22% (Fig. 5*E*,*F*). Notably, kainate also increased the mean coherence of GBO power between CA1 and CA3 by ∼15% (Fig. 5*G*). To visualize the variability of GBO power coherence within each brain section, we plotted pair-wise comparisons of GBO coherence per slice (Fig. 5*G*) and fitted the results to a linear mixed model (Table 1). Consistent with changes observed in mean GBO power coherence within each brain slice, the results from the linear mixed model indicated that kainate induced more robust increases in GBO coherence within the CA1 and CA3 subfields (*p* = 0.225) compared with between the subfields (Fixed effect coefficient −0.104; *p* < 0.0001; Fig. 5*H*). These data demonstrate that kainate application induces synchronization of GBOs within and between hippocampal subfields, with more robust coherence enhancements observed within the CA1 and CA3 subfields compared with between them.

### Schaffer collaterals play a key role in GBO synchronization between CA3 and CA1 hippocampal subfields

The Schaffer collaterals serve as pathways through which CA3 neurons project to CA1 neurons, connecting anatomically distant neuronal networks via a single excitatory synaptic connection (Amaral and Witter, 1989). This unilateral circuit enables CA3 excitatory projection neurons to modulate the activity of CA1 neurons, particularly within the frequencies of slow γ (∼25–50 Hz; Combe et al., 2018). However, while focal application of kainate can induce GBOs in CA1 (Chittajallu et al., 2013), it has been shown that CA1 GBOs can exist independently of CA3 GBOs (Craig and McBain, 2015).

**Figure 6.**
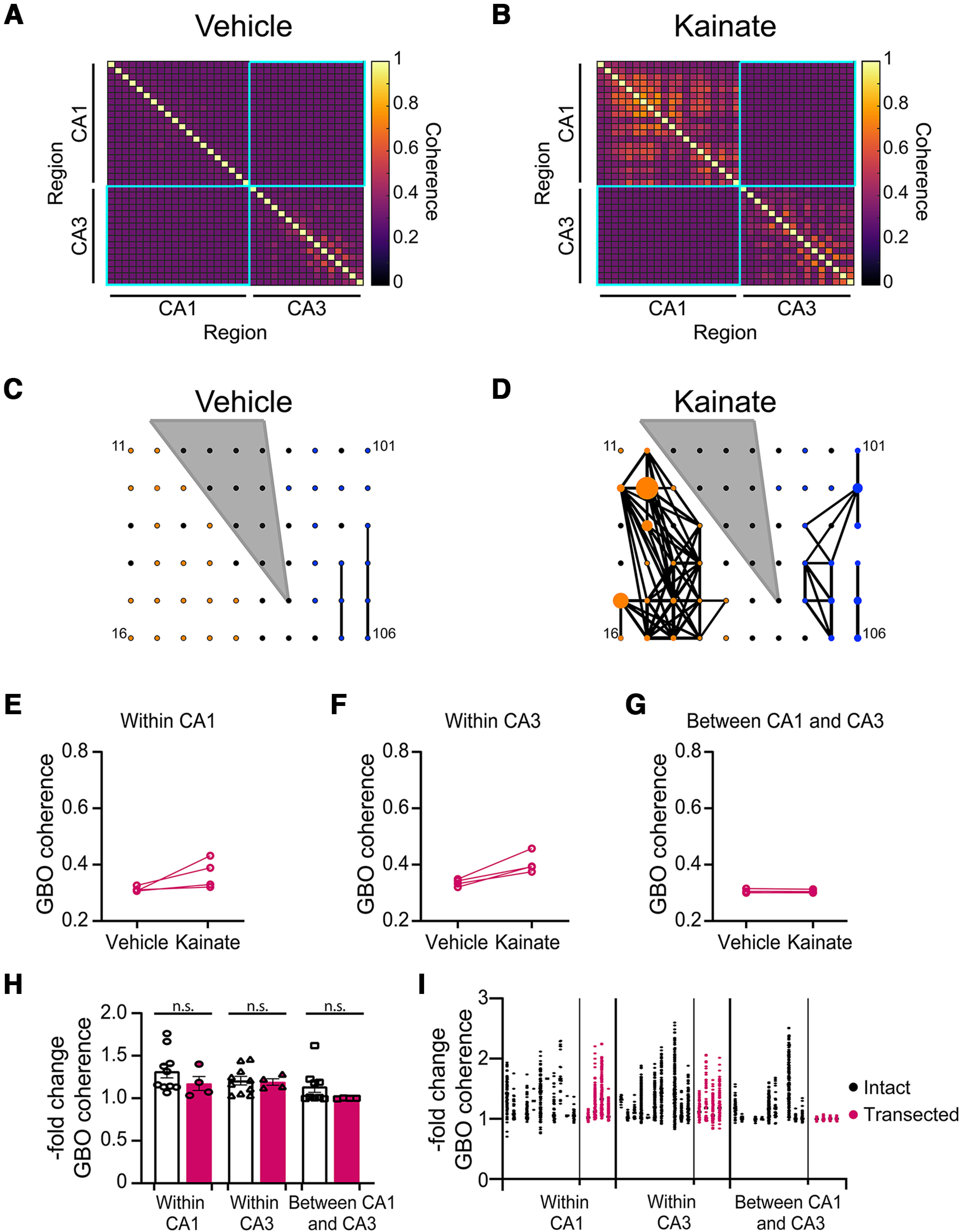
Schaffer collaterals mediate GBO coherence between, but not within CA1 and CA3. ***A***, ***B***, Representative correlation map of GBO power hippocampal sections with transected Schaffer collaterals in vehicle (***A***) or kainate (***B***). ***C***, ***D***, Representative graphical depiction of spatial GBO power coherence in hippocampal sections with transected Schaffer collaterals in vehicle (***C***) or kainate (***D***) gray triangle demarcates transections. Electrodes located in CA3 or CA1 are represented by blue and orange circles, respectively. The diameter is proportional to GBO power. Lines connect electrodes with GBO coherence >0.45. Mean GBO coherence within in CA1 (***E***) vehicle: 0.3126 ± 0.005; kainate: 0.368 ± 0.03 (*n *=* *4; *p *=* *0.125); in CA3 (***F***) vehicle: 0.336 ± 0.006; kainate: 0.4045 ± 0.02 (*n *=* *4, *p *=* *0.125); between CA1 and CA3 (***G***) vehicle: 0.3053 ± 0.004, kainate: 0.305 ± 0.003 (*n* = 4; *p *=* *0.125). Data shown as mean ± SEM, Wilcoxon test. ***H***, Kainate-evoked -fold change in mean GBO coherence within CA1 (intact: 1.132 ± 0.08, *n* = 10; transected: 1.173 ± 0.08, *n* = 4), within CA3 (intact: 1.207 ± 0.05, *n* = 10; transected: 1.191 ± 0.04, *n *=* *4); or between CA1 and CA3 (intact: 1.137 ± 0.07, *n* = 9; transected: 0.999 ± 0.002, *n *=* *4; Kruskal–Wallis statistic: 13.39, *p *=* *0.020; Dunn’s multiple comparison for: within CA1 intact vs transected *p* ≥ 0.999; within CA3 intact vs transected *p* ≥ 0.999; between CA1 and CA3 intact vs transected *p* = 0.260). Data shown as mean ± SEM, *n* = number of slices. ***I***, Kainate-evoked -fold change in GBO coherence per electrode, grouped by hippocampal subfield. Each circle represents a paired comparison of GBO power between two electrodes in hippocampal sections with intact (black) or transected Schaffer collaterals (magenta). See Table 2 for results from linear mixed effects model Panels ***J*** and ***K*** from Extended Data Figure 6-1 shows quantitative characterization of kainate-evoked GBOs from hippocampal sections with intact or transected Schaffer collaterals. Panel ***J*** shows fold change in GBO power in CA1 or CA3 from hippocampal sections with intact Schaffer collaterals (CA1: 1.313 ± 0.116, *N *=* *11, *n *=* *93; CA3: 1.651 ± 0.146, *N *=* *11, *n *=* *155), or transected Schaffer collaterals (CA1: 1.637 ± 0.182, *N *=* *4, *n *=* *71; CA3: 1.120 ± 0.151, *N *=* *4, *n *=* *59; CA1 vs CA1 transected *p* = 0.297; CA3 vs CA3 transected *p *=* *0.085). Panel ***K*** shows peak GBO frequency in CA1 (intact: 30.0 ± 1.2 Hz, *N *=* *11, *n *=* *93; transected: 21.0 ± 2.2 Hz, *N *=* *4, *n *=* *71). Peak GBO frequency in CA3 (intact: 21.13 ± 1.0 Hz, *N *=* *11, *n *=* *155; transected: 7.8 ± 0.8 Hz, *N *=* *4, *n *=* *59). Outliers were identified and removed using the ROUT method with a *Q *=* *0.1%.

To investigate the mechanism underlying slow GBO coherence between CA3 and CA1 γ, we conducted experiments in which we transected the Schaffer collaterals. The power of kainate-evoked GBOs was similar in in hippocampal sections with transected Schaffer collaterals, but peak frequency was reduced (Extended Data Fig. 6-1). The mean GBO coherence detected between electrodes within the CA1 or CA3 subfields of the transected hippocampal sections increased by ∼17% or 19%, respectively (Fig. 6*B*,*D–F*).

10.1523/ENEURO.0167-23.2023.f6-1 Extended Data Figure 6-1 Characteristics of GBOs in hippocampal slices with intact or transected Schaffer collaterals (SC). ***A***, ***B***, Quantitative characterization of kainate-evoked GBOs from hippocampal sections with intact or transected Schaffer collaterals. ***A***, Fold change in GBO power in CA1 or CA3 from hippocampal sections with intact Schaffer collaterals (CA1: 1.313 ± 0.116, *N *=* *11, *n *=* *93; CA3: 1.651 ± 0.146, *N *=* *11, *n *=* *155); or transected Schaffer collaterals (CA1: 1.637 ± 0.182, *N *=* *4, *n *=* *71; CA3: 1.120 ± 0.151, *N *=* *4, *n *=* *59). ***B***, Peak GBO frequency in CA1 (intact: 30.06 ± 1.2 Hz, *N *=* *11, *n *=* *93; transected: 21.0 ± 2.2 Hz, *N *=* *4, *n *=* *71. Peak GBO frequency in CA3 (intact: 21.13 ± 1.0 Hz, *N *=* *11, *n *=* *155; transected: 7.8 ± 0.8 Hz, *N *=* *4, *n *=* *59). Outliers were identified and removed utilizing the ROUT method with a Q = 0.1%. Download Figure 6-1, TIF file

**Table 2:** Linear mixed effect model of GBO coherence in lesioned hippocampal sections

Linear mixed-effects model fit by ML								
Model information:								
Number of observations	5435							
Fixed effects coefficients	6							
Random effects coefficients	378							
Covariance parameters	3							

Formula:								
Coherence ∼ 1 + Transected*Regions + (1 | Slice) + (1 | Slice:Elec1)								

Model fit statistics:								
AIC	BIC	LogLikelihood	Deviance					
−3549.6	−3490.2	1783.8	−3567.6					

Fixed effects coefficients (95% CIs):								
Name	Estimate	SE	*t* stat	df	*p* value	Lower	Upper	
‘(Intercept)’	1.231	0.047	26.1	5429	<0.001	1.139	1.324	
‘Transected_1’	0.041	0.090	-	0.5	5429	0.653	−0.218	0.136
‘Regions_CA1’	0.030	0.023	1.3	5429	0.186	−0.015	0.076	
‘Regions_CA1_3’	0.104	0.007	−15.4	5429	<0.001	−0.118	−0.091	
‘Transected_1:								
Regions_CA1’	-	0.028	0.035	−0.8	5429	0.419	−0.097	0.040
‘Transected_1:								
Regions_CA1_3’	−0.090	0.012	−7.5	5429	<0.001	−0.114	−0.067	

Random effects covariance parameters (95% CIs):								
Group: Slice (15 Levels)								
Name1	Name2	Type	Estimate	Lower	Upper			
(Intercept)’	‘(Intercept)’	‘std’	0.149	0.103	0.217			

Group: Slice:Elec1 (363 Levels)								
Name1	Name2	Type	Estimate	Lower	Upper			
‘(Intercept)’	‘(Intercept)’	‘std’	0.129	0.119	0.141			

Group: Error								
Name	Estimate	Lower	Upper					
‘Res Std’	0.162	0.158	0.165					

**Table 3:** Linear mixed effect model of GBO coherence in hippocampal sections from *Ank3* mouse

Linear mixed-effects model fit by ML							
Model information:							
Number of observations	2595						
Fixed effects coefficients	6						
Random effects coefficients	221						
Covariance parameters	3						

Formula:							
Coherence ∼ 1 + Model*Regions + (1 | Slice) + (1 | Slice:Elec1)							

Model fit statistics:							
AIC	BIC	LogLikelihood	Deviance				
−131.39	−78.641	74.697	−149.39				

Fixed effects coefficients (95% CIs):							
Name	Estimate	SE	*t* stat	df	*p* value	Lower	Upper
‘(Intercept)’	1.290	0.073	17.7	2589	<0.001	1.148	1.434
‘Model_1’	0.079	0.103	0.8	2589	0.440	−0.122	0.281
‘Regions_CA1’	−0.008	0.034	−0.2	2589	0.822	−0.074	0.058
‘Regions_CA1_3’	−0.244	0.022	−11.2	2589	<0.001	−0.287	−0.201
‘Model_1:Regions_CA1’	−0.271	0.043	−6.3	2589	<0.001	−0.356	−0.187
‘Model_1:Regions_CA1_3’	−0.176	0.027	−6.6	2589	<0.001	−0.229	−0.124

Random effects covariance parameters (95% CIs):							
Group: Slice (10 Levels)							
Name1	Name2	Type	Estimate	Lower	Upper		
‘(Intercept)’	‘(Intercept)’	‘std’	0.156	0.099	0.247		

Group: Slice:Elec1 (211 Levels)							
Name1	Name2	Type	Estimate	Lower	Upper		
‘(Intercept)’	‘(Intercept)’	‘std’	0.099	0.085	0.114		

Group: Error							
Name	Estimate	Lower	Upper				
‘Res Std’	0.223	0.217	0.230				

As expected, transecting the Schaffer collaterals prevented an increase in GBO coherence between electrodes in CA1 and CA3 (Fig. 6*H*). However, direct comparison of the mean GBO coherence between all possible pair-wise comparisons within CA1 and CA3 of control slices or transected slices did not yield statistical significance (Kruskal–Wallis *p* = 0.020; Dunn’s multiple comparisons within CA1 *p* ≥ 0.999; within CA3 *p* > 0.999; between CA1 and CA3 *p* = 0.260). It is important to note that comparing the means of averages between control and experimental groups can oversimplify the data by disregarding variability. To better understand the differences within the data and preserve variability, we employed a linear mixed model (Table 2). When we incorporated the Schaffer collaterals as a predictor variable in the linear mixed model, the results indicated that severing the Schaffer collaterals was not a predictor of kainate-induced changes in overall GBO coherence. However, severing the Schaffer collaterals was a strong predictor for the decrease in GBO coherence between CA1 and CA3 (*p* < 0.0001), providing support for the crucial role of Schaffer collaterals in synchronizing GBOs between the CA3 and CA1 subregions.

### Attenuated GBO power in *Ank3* mouse hippocampal sections

**Figure 7.**
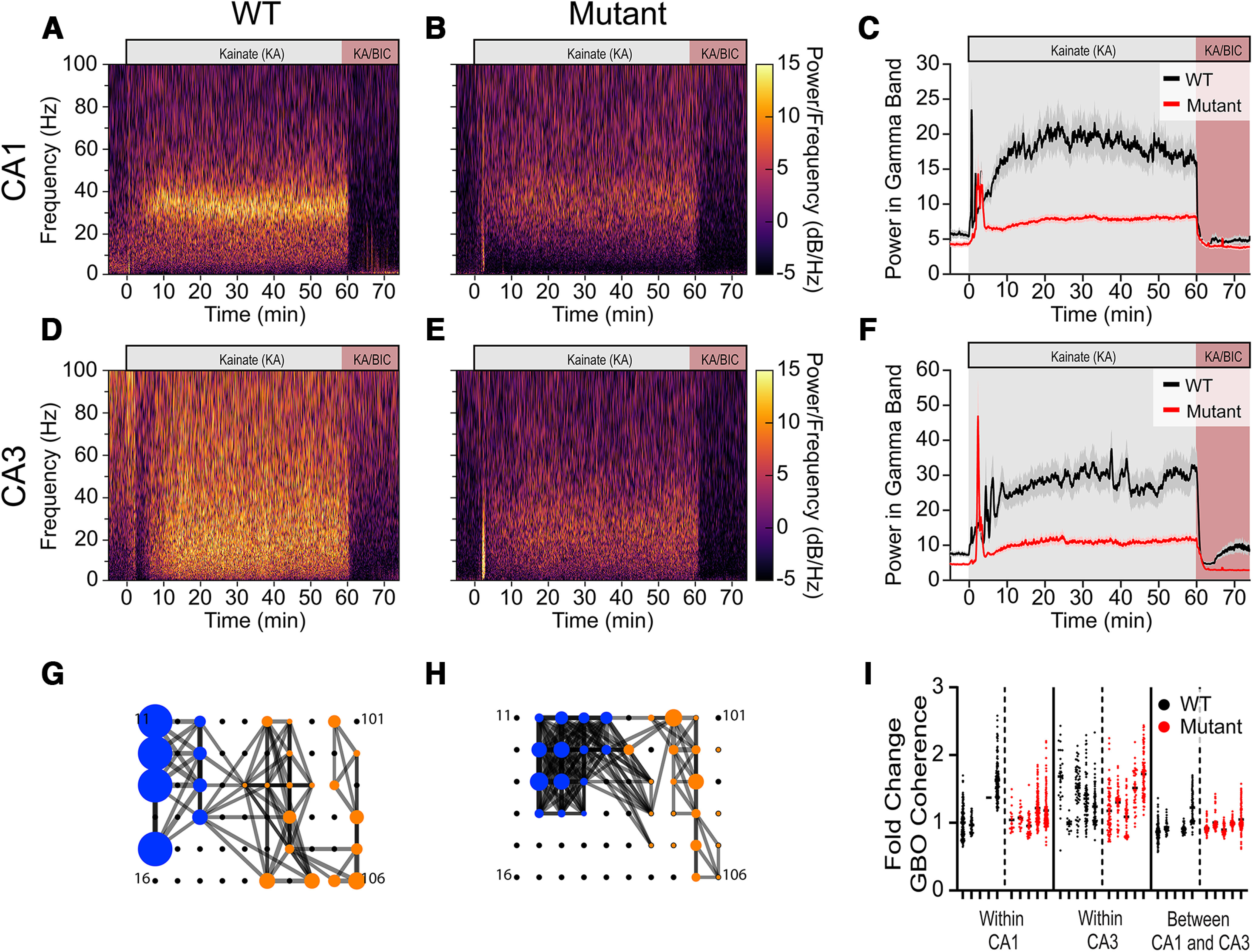
GBO power and intrasubfield coherence are attenuated in p.W1989R (*Ank3*) mutant mice. ***A***, ***B***, Representative power spectra in CA1 of WT (***A***) or *Ank3* mice (***B***). ***C***, GBO power for WT (black; *N *=* *4, *n* = 50) and *Ank3* mutant mice (red; *N *=* *5, *n *=* *62). ***D***, ***E***, Representative power spectra in CA3 of WT (***D***) or *Ank3* mice (***E***). ***F***, GBO power for WT mice (black; *N *=* *5, *n* = 50) and *Ank3* mice (red; *N *=* *5, *n *=* *59). Data shown as mean ± SEM. ***G***, ***H***, Spatial coherence map for WT (***G***) and *Ank3* mutant mice (***H***). Electrodes located in CA3 or CA1 are shown as blue or orange circles, respectively. The diameter is proportional to GBO power. Lines connect electrodes with GBO coherence >0.45 0.45 are connected by lines. Line density indicates the strength of coherence. ***I***, Fold change in coherence for each electrode comparison grouped in slices. Individual circles represent a correlation in GBO power between a pair of electrodes. Black circles represent WT and red circles represent mutant mice. See Table 3 for results from linear mixed effects model. Extended Data Figure 7-1 shows the power of GBOs of WT and MT mice in CA1 (WT: 16.59 ± 2.30 μV^2^, *N *=* *4, *n *=* *50 electrodes vs MT: 8.16 ± 0.57 μV^2^, *N *=* *5, *n *=* *62 electrodes; *p* = 0.007) and CA3 (WT: 30.45 ± 5.01 μV^2^, *N *=* *4, *n *=* *50 electrodes vs MT: 11.70 ± 1.26 μV^2^, *N *=* *5, *n *=* *62 electrodes; *p* = 0.002) after kainate induction. Extended Data Figure 7-2 shows the latency for GBOs to reach 90% of maximum for WT and *Ank3* mutant mice. The GBO latency in WT mice was 13.72 ± 0.92 min in CA3, *n *=* *45, *N *=* *4; and 12.85 ± 0.57 min in CA1, *n *=* *50, *N *=* *4. In *Ank3* mutant mice was 13.54 ± 0.74 min in CA3, *n *=* *57, *N *=* *4; and 15.09 ± 0.85 min, in CA1 *n* = 62, *N *=* *4 (Extended Data Figure 7-3 shows the 90–10 fall time for WT and MT mice. Fall time in CA1 was 46.92 ± 4.13 s, *n* = 47, *N* = 4, for WT mice; and 55.35 ± 6.61 s, *n* = 59, *N* = 5 for *Ank3* mutant mice (Kruskal–Wallis test 1.95; *p* = 0.582). Fall time in CA3 was 40.48 ± 3.45 s, *n* = 47, *N* = 5 for WT mice and 47.6165.45, *n* = 57, *N* = 5 for *Ank3* mutant mice. Panels ***A*** and ***B*** in Extended Data Figure 7-4 show representative maps of GBO coherence during bath application of kainate for WT and *Ank3* mutant mouse. Panel ***C*** in Extended Data Figure 7-4 shows mean GBO coherence between electrode pairs within CA1. WT vehicle: 0.41 ± 0.01, *N* = 5; *Ank3* vehicle: 0.40 ± 0.05, *N* = 4; WT kainate: 0.413 ± 0.01, *N* = 5; *Ank3* kainate: 0.51 ± 0.08, *N* = 4; two-way RM ANOVA Genotype X Phase *p* = 0.2641; Phase *p* = 0.0548; Genotype *p* = 0.1306). Panel ***D*** in Extended Data Figure 7-4 shows mean GBO coherence between electrode pairs within CA3. WT vehicle: 0.41 ± 0.01, *N* = 5; *Ank3* vehicle 0.360 ± 0.02, *N* = 5; WT kainate: 0.51 ± 0.03, *N* = 5; *Ank3* kainate 0.53 ± 0.04, *N* = 4; two-way RM ANOVA Genotype X Phase *p* = 0.9901; Phase *p* = 0.0020; Genotype *p* = 0.4906. Panel ***E*** in Extended Data Figure 7-4 shows mean GBO coherence of electrode pairs between CA1 and CA3. WT vehicle: 0.40 ± 0.01, *N* = 5; *Ank3* vehicle 0.33 ± 0.01, *N* = 4; WT kainate: 0.359 ± 0.009, *N* = 5; *Ank3* kainate 0.34 ± 0.01, *N* = 4; two-way RM ANOVA Genotype X Phase *p* = 0.9698; Phase *p* = 0.4348; Genotype *p* = 0.7785). Panel ***E*** in Extended Data Figure 7-4 shows -fold change in mean GBO coherence between electrodes during the last 5 min bath application of kainate within CA1 (WT: 1.01 ± 0.05, *N* = 5, *Ank3*: 1.18 ± 0.09, *N* =4; *p* = 0.334); within CA3 (WT: 1.24 ± 0.12, *N* = 5, *Ank3*: 1.47 ± 0.08, *N* = 5; *p* > 0.999); and between CA1 and CA3 (WT: 0.908 ± 0.02, *N* = 5, MT: 1.04 ± 0.06, *N* =4, *p* < 0.999). Kruskal–Wallis statistic = 20.53; *p* = 0.001. Data shown as mean ± SEM, *n* = number of electrodes, *N* = number of slices; **p < 0.01.

To showcase the utility of our approach, we investigated kainate-induced GBOs in hippocampal brain sections obtained from mice carrying a disease-associated variant in *Ank3* (Nelson et al., 2020). Ankyrins, a family of scaffolding proteins widely expressed throughout the body, play critical roles in neuronal structure and function (Bennett and Lorenzo, 2013; Nelson and Jenkins, 2017). Genome-wide association studies (GWAS) have linked *Ank3* with psychiatric disorders, including bipolar disorder (Stevens and Rasband, 2021). In a previous study, the disease-associated *Ank3* variant, *Ank3* p.W1989R, was shown to diminish GABAergic neurotransmission from fast-spiking interneurons (FSIs) and reduces the power of kainate-evoked GBOs in hippocampal sections from mice carrying this variant (Nelson et al., 2020). We hypothesized that impaired GABAergic inhibition resulting from the *Ank3* mutations would lead to a decreased capacity of hippocampal sections to generate and sustain GBOs. Consistent with WT littermates, the bath application of kainate promptly increased broadband LFP power in hippocampal sections from *Ank3* mutant mice, which subsequently stabilized into GBOs (Fig. 7*A*,*B*,*D*,*E*; Extended Data Fig. 7-2). However, we observed a reduction in the power of kainate-induced GBOs in *Ank3* mice (Fig. 7*C*,*F*; Extended Data Fig. 7-1), consistent with a previous report (Nelson et al., 2020). Notably, there were no discernible differences in the temporal onset of GBOs between the CA1 and CA3 subfields of hippocampal sections from *Ank3* mutant mice (Kruskal–Wallis statistic = 4.957, *p* = 0.175; Extended Data Fig. 7-2). Moreover, co-application of bicuculline rapidly abolished kainate-induced GBOs in hippocampal sections of *Ank3* mutants, following a similar time course as observed in WT littermates (Kruskal–Wallis statistic = 1.953, *p* = 0.582; Extended Data Fig. 7-3). Thus, in *Ank3* mutant mice, the power of kainate-induced GBOs in hippocampal sections was reduced, underscoring the impact of *Ank3* mutations on GBO generation. A portion of the data presented in Figure 7 was previously published in Figure 2 and Supplementary Figure 8 of Nelson et al. (2020).

10.1523/ENEURO.0167-23.2023.f7-1 Extended Data Figure 7-1 GBO power is attenuated in p.W1989R (*Ank3*) mutant mice. Power of GBOs of WT and MT mice in CA1 (WT: 16.59 ± 2.30 μV^2^, *N *=* *4, *n *=* *50 electrodes vs MT: 8.16 ± 0.57 μV^2^, *N *=* *5, *n *=* *62 electrodes; *p* = 0.007) and CA3 (WT: 30.45 ± 5.01 μV^2^, *N *=* *4, *n *=* *50 electrodes vs MT: 11.70 ± 1.26 μV^2^, *N *=* *5, *n *=* *62 electrodes; *p* = 0.002) after kainate induction. Data shown as mean ± SEM. *n* = number of electrodes, *N* = number of slicesl **p <* *0.01. Download Figure 7-1, TIF file.

10.1523/ENEURO.0167-23.2023.f7-2 Extended Data Figure 7-2 The latency of kainate-evoked GBOs is similar in CA1 and CA3. The latency for GBOs to reach 90% of maximum for WT mice was 13.72 ± 0.92 min in CA3, *n *=* *45, *N *=* *4; and 12.85 ± 0.57 min in CA1, *n *=* *50, *N *=* *4. In *Ank3* mutant mice, GBO latency was 13.54 ± 0.74 min in CA3, *n *=* *57, *N *=* *4; and 15.09 ± 0.85 min, in CA1 *n* = 62, *N *=* *4. Data shown as mean ± SEM. *n* = number of electrodes, *N* = number of slices. Download Figure 7-2, TIF file.

10.1523/ENEURO.0167-23.2023.f7-3 Extended Data Figure 7-3 Bicuculline abolished GBO in WT and MT mice with similar temporal kinetics. 90–10 fall time (time to reduce GBOs from 90% to 10% of max) was not different between WT and MT mice (Kruskal–Wallis test 1.95; *p* = 0.582). Fall time for CA1 in WT was 46.92 ± 4.13 s, *n* = 47, *N* = 4; while for MT, it was 55.35 ± 6.61 s, *n* = 59, *N* = 5. Fall time for CA3 was 40.48 ± 3.45, *n* = 47, *N* = 5; and 47.61 ± 5.45, *n* = 57, *N* = 5 for WT and MT, respectively. Data shown as mean ± SEM. *n* = number of electrodes, *N* = number of slices. Download Figure 7-3, TIF file.

10.1523/ENEURO.0167-23.2023.f7-4 Extended Data Figure 7-4 Coherence in Ank3 mouse model. ***A***, ***B***, Representative map of coherence during bath application of kainate for (***A***) WT and (***B***) mutant mouse. *C–E*, Mean coherence during the last 5 min of vehicle and during kainate for electrode pairs (***C***) within CA1 (WT vehicle: 0.41 ± 0.01, *N* = 5; MT 0.40 ± 0.05, *N* = 4; kainate: 0.413 ± 0.01, *N* = 5; MT 0.51 ± 0.08, *N* = 4; two-way RM ANOVA Genotype × Phase *p* = 0.2641; Phase *p* = 0.0548; Genotype *p* = 0.1306), (***D***) within CA3 (WT vehicle: 0.41 ± 0.01, *N* = 5; MT 0.360 ± 0.02, *N* = 5; kainate: 0.51 ± 0.03, *N* = 5; MT 0.53 ± 0.04, *N* = 4; two-way RM ANOVA Genotype × Phase *p* = 0.9901; Phase *p* = 0.0020; Genotype *p* = 0.4906), and (***E***) between CA1 and CA3 (WT vehicle: 0.40 ± 0.01, *N* = 5; MT 0.33 ± 0.01, *N* = 4; kainate: 0.359 ± 0.009, *N* = 5; MT 0.34 ± 0.01, *N* = 4; two-way RM ANOVA Genotype × Phase *p* = 0.9698; Phase *p* = 0.4348; Genotype *p* = 0.7785). ***F***, Fold change in mean coherence from vehicle to kainate (Kruskal–Wallis statistic = 20.53; *p* = 0.001) within CA1 (WT: 1.01 ± 0.05, *N* = 5, MT: 1.18 ± 0.09, *N* = 4; *p* = 0.334), within CA3 (WT: 1.24 ± 0.12, *N* = 5, MT: 1.47 ± 0.08, *N* = 5; *p* > 0.999) and between CA1 and CA3 (WT: 0.908 ± 0.02, *N* = 5, MT: 1.04 ± 0.06, *N* = 4, *p* > 0.999). Data shown as mean ± SEM, *N* = number of slices. Kruskal–Wallis test and Dunn’s tests for multiple comparisons were performed in GraphPad Prism. Download Figure 7-4, TIF file.

### GBO coherence is impaired in CA1 but not CA3 of *Ank3* mice

GBO coherence in *Ank3* mice showed comparable robustness to that observed in hippocampal sections from WT littermates (Fig. 7*A–F*; Extended Data Fig. 7-4). Under basal conditions, mean GBO coherence within and between all hippocampal subregions did not differ significantly between WT and *Ank3* mice: within CA1 (WT: 0.41 ± 0.01, *N* = 5; *Ank3*: 0.40 ± 0.05, *N* = 4; Extended Data Fig. 7-4*C*), within CA3 (WT: 0.41 ± 0.01, *N* = 5; *Ank3*: 0.360 ± 0.02, *N* = 5; Extended Data Fig. 7-4*D*), between CA1 and CA3 (WT: 0.40 ± 0.01, *N* = 5; *Ank3*: 0.33 ± 0.01, *N* = 4; Extended Data Fig. 7-4*E*). Spatial mapping of electrodes revealed that kainate application broadly increased GBO coherence across most electrodes localized in a hippocampal slice from an *Ank3* mutant mouse (Fig. 7*G*,*H*). The mean GBO coherence within and between hippocampal subfields differed between sections taken from *Ank3* MT mice and WT littermates (Kruskal–Wallis statistic = 20.53; *p* = 0.0010). However, multiple comparison tests demonstrated that mean GBO coherence within CA3 (*p* > 0.999), within CA1 (*p* = 0.334) and between CA3 and CA1 (*p* > 0.999) did not differ (Extended Data Fig. 7-4*F*). We incorporated mouse genotype as a predictor variable in a linear mixed effects model to account for variance within the coherence measurements (Fig. 7*I*). The model revealed that the *Ank3* mutation itself was not a significant predictor (*p* = 0.434). However, the mutation exhibited interactions within CA1 region coherence (*p* < 0.0001) and between CA1 and CA3 coherence (*p* < 0.0001; Table 3). Taken together, these findings imply that the reduced GABAergic inhibition caused by the *Ank3* mutant does not impair the capacity to generate GBOs, but does significantly reduce GBO power. We previously demonstrated that the dysfunction of parvalbumin-expressing interneurons contributed to the decreased inhibitory neurotransmission and altered hippocampal GBOs in *Ank3* mutant mice (Nelson et al., 2020). Thus, although the overall power of GBOs is reduced, the coherence of GBOs remains intact within CA3 of *Ank3* mice, but attenuated within CA1 and between CA3 and CA1. This suggests that deficits to parvalbumin-expressing interneurons caused by the *Ank3* mutation may specifically impair mechanisms of GBO coherence within and between the CA1 subfield.

## Discussion

In this study, we have successfully developed a cutting-edge approach for investigating kainate-evoked γ-band oscillations (GBOs) in *ex vivo* hippocampal slices. Our method showcases the ability to rapidly generate, sustain, and pharmacologically modulate GBOs in mature mouse hippocampal sections for extended durations of over 90 min. Leveraging multilocation LFP recordings within hippocampal subregions, we harnessed computational algorithms to precisely quantify GBO coherence within and between the CA1 and CA3 hippocampal subfields. To validate the broad applicability of our approach, we employed a transgenic mouse model with inhibitory synaptic dysfunction, effectively demonstrating how our methodology can uncover comprehensive insights into generalized deficits in oscillatory activity. Notably, our findings not only substantiate previous observations of reduced GBO power in *Ank3* mutant mice but also unveil nuanced impairments in the synchronization of GBOs within and between the hippocampal subfields of *Ank3* mice.

### GBOs elicited by kainate unveil glutamatergic activation pathway

In our investigation, we induced γ-band oscillations (GBOs) by employing a bath application of kainate, a potent glutamatergic agonist. It is important to note that GBOs can also be elicited in *ex vivo* hippocampal sections through various pharmacological manipulations, including the use of cholinergic agonists (Fisahn et al., 1998). The specific activation of GBOs by kainate is mediated by the engagement of GluK5 and GluK6 receptors (Fisahn et al., 2004), which are believed to be predominantly expressed by GABAergic interneurons within the hippocampus (Paternain et al., 2000).

### Kainate and cholinergic agonists elicitation GBOs by distinct mechanisms

In contrast, the activation of GBOs by cholinergic agonists occurs through the stimulation of M1 receptors (Fisahn et al., 1998), which are equally expressed by both pyramidal neurons and GABAergic interneurons in the hippocampus (Cea-del Rio et al., 2010). Notably, kainate is known to elicit more robust GBOs (Cunningham et al., 2003), whereas cholinergic agonists tend to induce GBOs with higher peak frequencies (Fisahn et al., 1998). These findings suggest that kainate and carbachol evoke GBOs through partially distinct mechanisms. However, it is important to acknowledge that direct comparisons of results obtained from different studies can be challenging because of variations in experimental setups, such as differences in apparatuses, perfusion kinetics, and local oxygen partial pressures. To gain further insights and distinguish the underlying mechanisms, it would be beneficial to characterize the temporal dynamics of GBO coherence specifically between kainate-induced and carbachol-induced GBOs under consistent experimental conditions, similar to those employed in our study. The significant progress achieved in this study was largely driven by two crucial technological advancements.

### pMEAs facilitate the robust generation and modulation of GBOs in *ex vivo* tissue

The utilization of pMEA technology is the first key technological advancement in our approach. pMEAs played a pivotal role in reliably evoking GBOs in *ex vivo* brain sections by addressing the energetic demands of these oscillations that require near-maximal mitochondrial oxidative capacity (Kann et al., 2011). By enhancing the delivery of oxygenated aCSF to the hippocampal sections and increasing the local O_2_ partial pressure within the interstitial space (Egert et al., 2005), pMEAs ensured consistent generation of GBOs and facilitated their pharmacological modulation (Figs. 3, 4). Additionally, the density and geometry of the electrode arrays employed in our study enabled simultaneous recordings from >10 electrodes in each hippocampal subfield, significantly enhancing spatial resolution. In contrast, a previous study on kainate-induced GBOs only recorded LFPs from a single electrode placed in each CA1 and CA3 subfield (C. Lu et al., 2012). This improved spatial resolution enabled us to investigate the temporal development of GBO coherence within and between CA1 and CA3. By selectively severing the Schaffer collaterals, the axonal projections of CA3 pyramidal cells that innervate the apical dendrites of CA1 pyramidal cells or CA1 parvalbumin-expressing FSIs, we discovered that kainate independently induces GBOs within CA1 and CA3 subfields while eliminating power-power coherence between them. Our interpretation of these findings suggests that CA1 and CA3 possess distinct mechanisms for generating kainate-induced GBOs, with the synchronization of GBOs between subfields dependent on intact Schaffer collaterals. Notably, empirical studies (Colgin et al., 2009) and computational models (Mysin et al., 2019) support these interpretations, reinforcing the robustness and validity of our conclusions.

### Enhanced viability and age flexibility in generating GBOs through NMDG recovery

Another significant advancement in our approach was the incorporation of the NMDG recovery method, which expanded the age range of mice from which GBOs could be continuously evoked. Typically, GBOs are studied using *ex vivo* brain sections from young animals (Carmeli et al., 2013; Hájos et al., 2009; Tsintsadze et al., 2015). Although there are reports of GBOs evoked in hippocampal sections from older mice, the ability to evoke GBOs is often transient (Gloveli et al., 2005), making *ex vivo* tissue from neonatal animals more viable for these experiments.

Generating GBOs *ex vivo* requires the presence of healthy fast-spiking interneurons (FSIs) and maintaining tissue viability throughout the experiment is crucial. By incorporating the NMDG recovery method into our approach, we significantly enhanced the viability of our brain sections (Ting et al., 2014). Importantly, the combination of pMEAs and NMDG recovery allowed us to evoke GBOs from *ex vivo* hippocampal sections across a broader range of developmental ages when FSIs are physiologically mature (Goldberg et al., 2011). This expanded age flexibility has valuable implications, particularly for studying GBOs in animal models of diseases that manifest later in life, such as schizophrenia or dementia. The utilization of the NMDG recovery method opens up new avenues for investigating oscillatory dynamics in more relevant disease models and deepening our understanding of the underlying mechanisms.

Our approach proved instrumental in uncovering previously unrecognized deficits in hippocampal GBOs by investigating a mouse model with impaired GABAergic neurotransmission. Previous research demonstrated reduced power in kainate-induced GBOs in *Ank3* mice (Nelson et al., 2020). In this study, we not only confirmed and expanded on those findings but also revealed that while kainate-induced GBO power is diminished in both the CA1 and CA3 regions of *Ank3* mice, GBO coherence is specifically attenuated in CA1. Although the mechanistic basis for the divergent GBO responses between CA1 and CA3 in *Ank3* mice requires further elucidation, it is plausible that the deleterious impact of the *Ank3* mutation primarily affects the mechanics of GBO coherence within CA1, while sparing CA3, given the partially distinct mechanisms underlying GBO generation in these regions (Combe et al., 2018).

In summary, we have presented a novel assay that successfully generates and sustains GBOs in *ex vivo* tissue from juvenile mice. This approach holds promise for characterizing a wide range of oscillatory frequencies in *ex vivo* sections from various excitable tissues and animal models. Moreover, we propose that the utility of this approach can be significantly expanded by incorporating focalized methods such as electrical, optogenetic, or chemogenetic stimulation to precisely generate oscillations. By leveraging these advancements, we can further unravel the complexities of oscillatory dynamics and deepen our understanding of their functional implications in health and disease.

## Synthesis

Reviewing Editor: Jibran Khokhar, University of Guelph Decisions are customarily a result of the Reviewing Editor and the peer reviewers coming together and discussing their recommendations until a consensus is reached. When revisions are invited, a fact-based synthesis statement explaining their decision and outlining what is needed to prepare a revision will be listed below. The following reviewer(s) agreed to reveal their identity: NONE. Note: If this manuscript was transferred from JNeurosci and a decision was made to accept the manuscript without peer review, a brief statement to this effect will instead be what is listed below.

This is a well designed and executed study that aimed to develop a novel approach to study gamma band oscillations in ex vivo hippocampal slices from mature animals. They used perforated microelectrode arrays and assessed gamma band synchrony, which was disrupted by transection of Schaffer collaterals and in Ank3 mutant mice. While the primary concern for the reviewers at JNeurosci were the descriptive and not mechanistic nature of the experiments, I found that the authors did a good job of addressing the reviewer comments and think that the paper is acceptable as is for eNeuro.
