# Supplementary figures and images for: A Novel Approach to Study Coherent γ-Band Oscillations in Hippocampal Brain Sections
Source: eNeuro. 2023 Jul 21;10(7):ENEURO.0167-23.2023. doi: 10.1523/ENEURO.0167-23.2023 (PMC10368148; doi:10.1523/ENEURO.0167-23.2023)

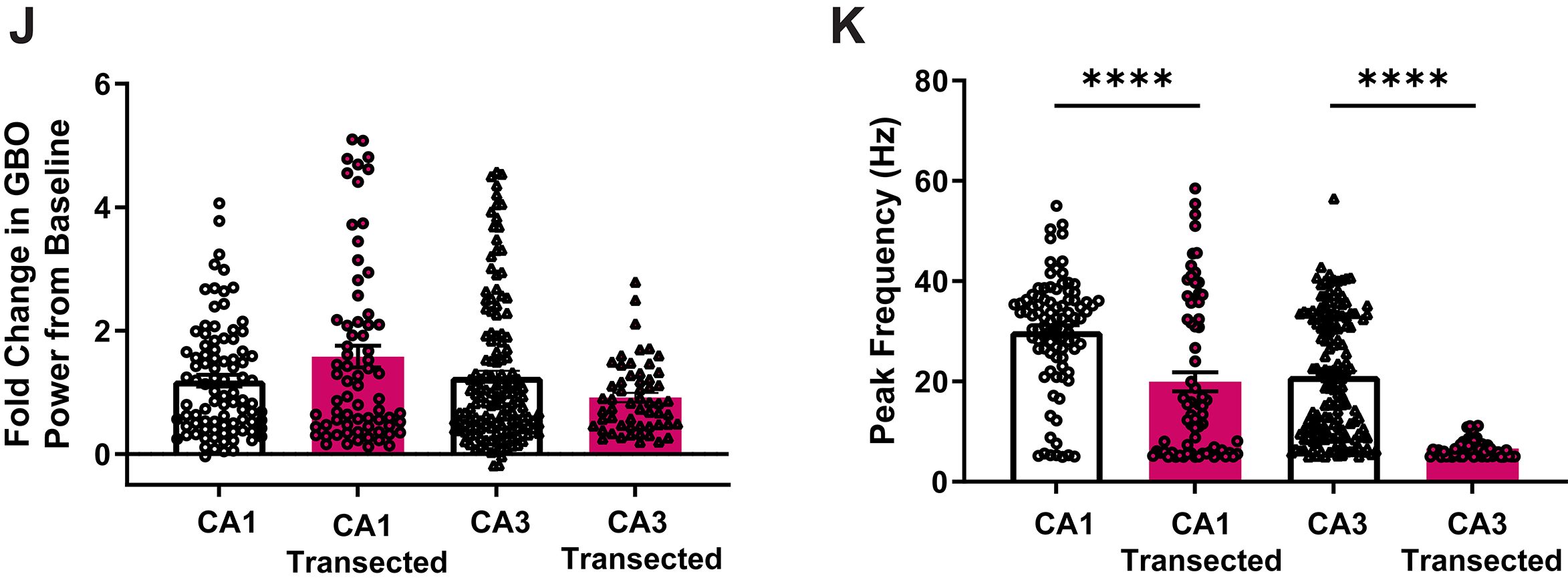

Supplement: Extended Data Figure 6-1 — Characteristics of GBOs in hippocampal slices with intact or transected Schaffer collaterals (SC). A, B, Quantitative characterization of kainate-evoked GBOs from hippocampal sections with intact or transected Schaffer collaterals. A, Fold change in GBO power in CA1 or CA3 from hippocampal sections with intact Schaffer collaterals (CA1: 1.313 ± 0.116, N = 11, n = 93; CA3: 1.651 ± 0.146, N = 11, n = 155); or transected Schaffer collaterals (CA1: 1.637 ± 0.182, N = 4, n = 71; CA3: 1.120 ± 0.151, N = 4, n = 59). B, Peak GBO frequency in CA1 (intact: 30.06 ± 1.2 Hz, N = 11, n = 93; transected: 21.0 ± 2.2 Hz, N = 4, n = 71. Peak GBO frequency in CA3 (intact: 21.13 ± 1.0 Hz, N = 11, n = 155; transected: 7.8 ± 0.8 Hz, N = 4, n = 59). Outliers were identified and removed utilizing the ROUT method with a Q = 0.1%. Download Figure 6-1, TIF file [file enu-eN-MNT-0167-23-s01.tif]

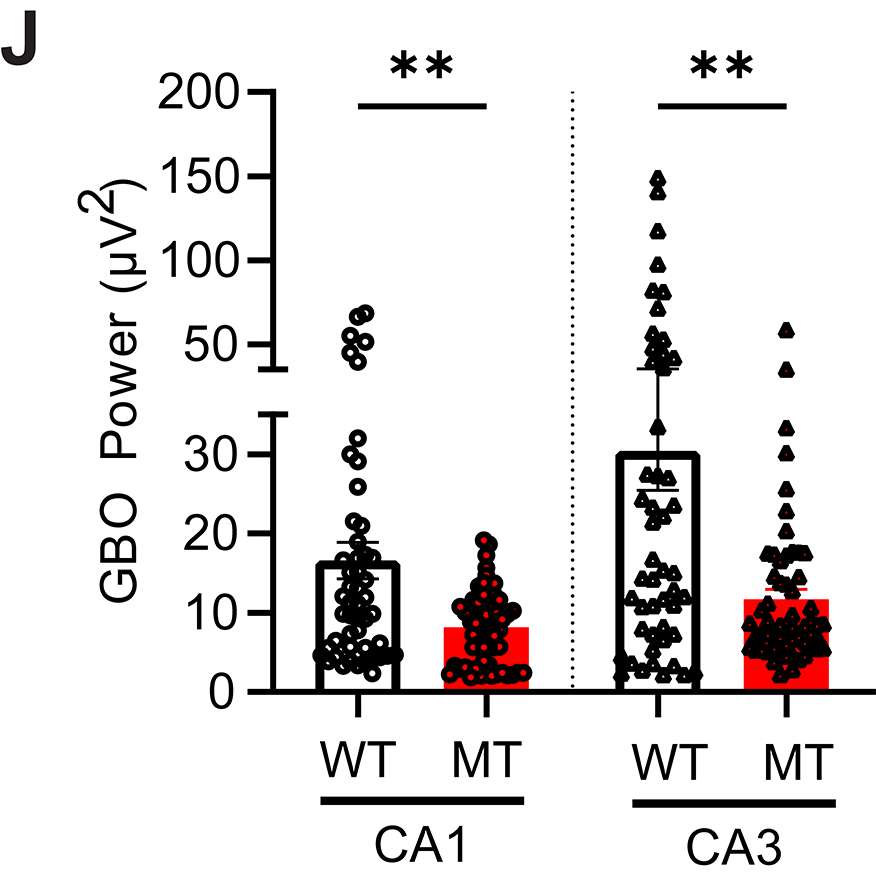

Supplement: Extended Data Figure 7-1 — GBO power is attenuated in p.W1989R (Ank3) mutant mice. Power of GBOs of WT and MT mice in CA1 (WT: 16.59 ± 2.30 μV2, N = 4, n = 50 electrodes vs MT: 8.16 ± 0.57 μV2, N = 5, n = 62 electrodes; p = 0.007) and CA3 (WT: 30.45 ± 5.01 μV2, N = 4, n = 50 electrodes vs MT: 11.70 ± 1.26 μV2, N = 5, n = 62 electrodes; p = 0.002) after kainate induction. Data shown as mean ± SEM. n = number of electrodes, N = number of slicesl **p < 0.01. Download Figure 7-1, TIF file. [file enu-eN-MNT-0167-23-s02.tif]

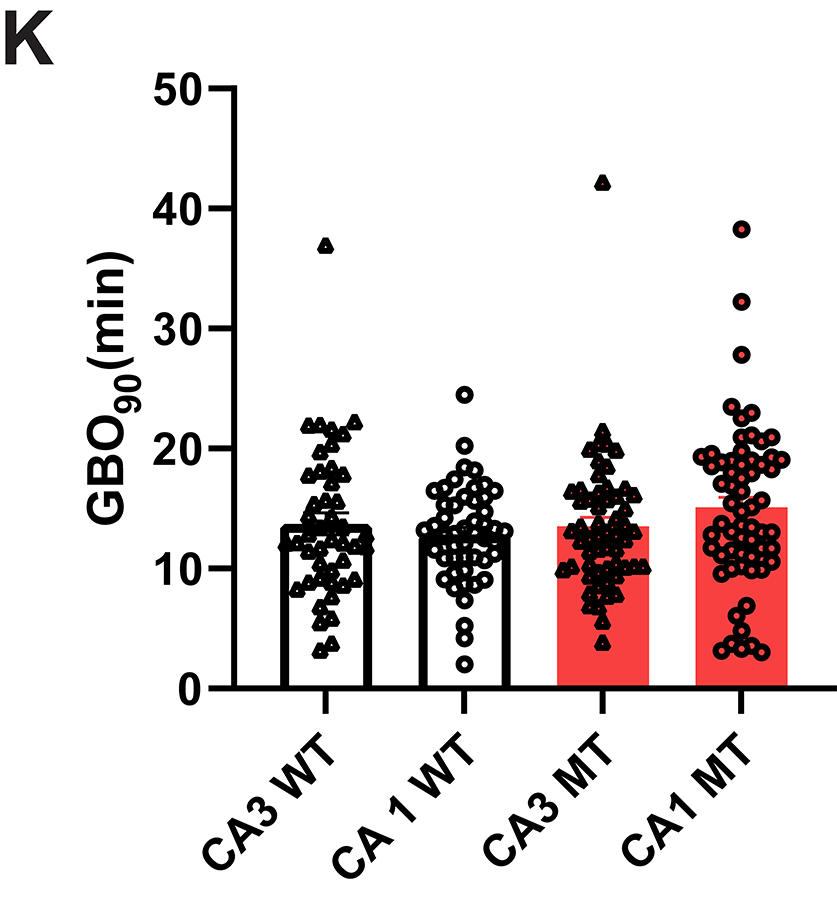

Supplement: Extended Data Figure 7-2 — The latency of kainate-evoked GBOs is similar in CA1 and CA3. The latency for GBOs to reach 90% of maximum for WT mice was 13.72 ± 0.92 min in CA3, n = 45, N = 4; and 12.85 ± 0.57 min in CA1, n = 50, N = 4. In Ank3 mutant mice, GBO latency was 13.54 ± 0.74 min in CA3, n = 57, N = 4; and 15.09 ± 0.85 min, in CA1 n = 62, N = 4. Data shown as mean ± SEM. n = number of electrodes, N = number of slices. Download Figure 7-2, TIF file. [file enu-eN-MNT-0167-23-s03.tif]

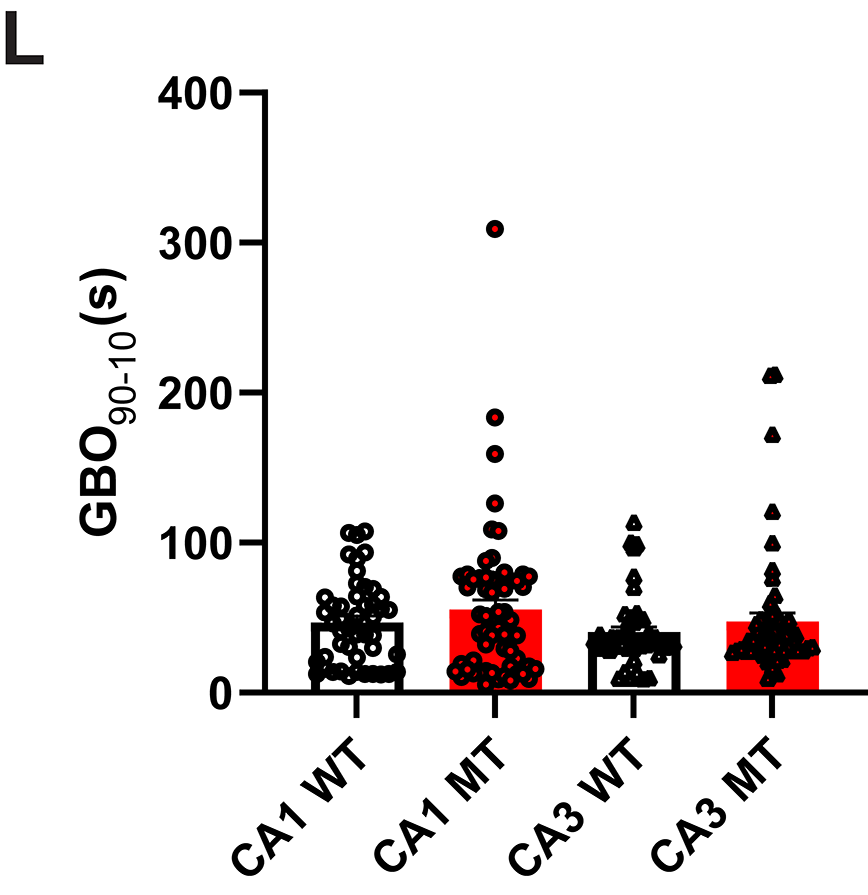

Supplement: Extended Data Figure 7-3 — Bicuculline abolished GBO in WT and MT mice with similar temporal kinetics. 90–10 fall time (time to reduce GBOs from 90% to 10% of max) was not different between WT and MT mice (Kruskal–Wallis test 1.95; p = 0.582). Fall time for CA1 in WT was 46.92 ± 4.13 s, n = 47, N = 4; while for MT, it was 55.35 ± 6.61 s, n = 59, N = 5. Fall time for CA3 was 40.48 ± 3.45, n = 47, N = 5; and 47.61 ± 5.45, n = 57, N = 5 for WT and MT, respectively. Data shown as mean ± SEM. n = number of electrodes, N = number of slices. Download Figure 7-3, TIF file. [file enu-eN-MNT-0167-23-s04.tif]

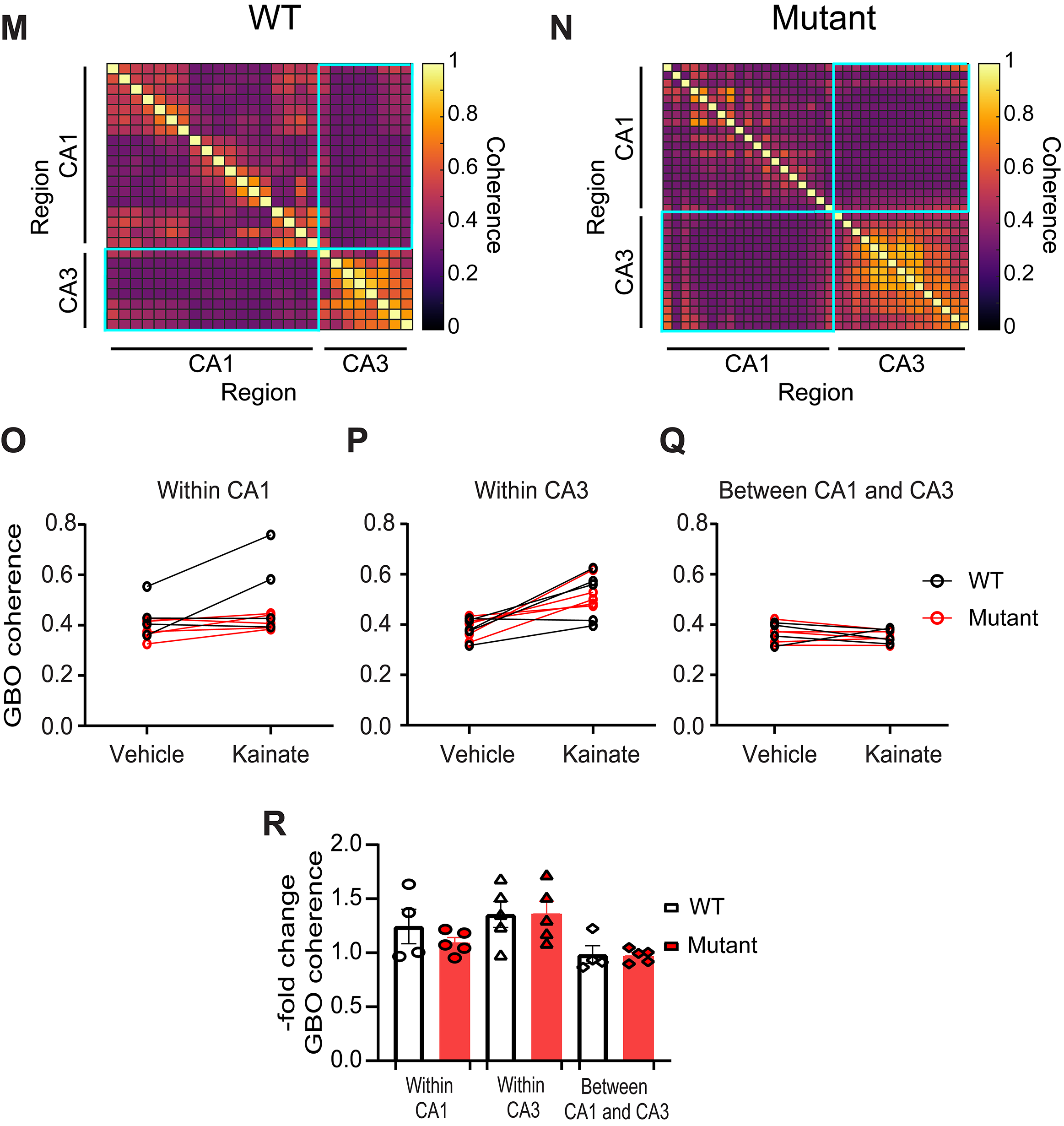

Supplement: Extended Data Figure 7-4 — Coherence in Ank3 mouse model. A, B, Representative map of coherence during bath application of kainate for (A) WT and (B) mutant mouse. C–E, Mean coherence during the last 5 min of vehicle and during kainate for electrode pairs (C) within CA1 (WT vehicle: 0.41 ± 0.01, N = 5; MT 0.40 ± 0.05, N = 4; kainate: 0.413 ± 0.01, N = 5; MT 0.51 ± 0.08, N = 4; two-way RM ANOVA Genotype × Phase p = 0.2641; Phase p = 0.0548; Genotype p = 0.1306), (D) within CA3 (WT vehicle: 0.41 ± 0.01, N = 5; MT 0.360 ± 0.02, N = 5; kainate: 0.51 ± 0.03, N = 5; MT 0.53 ± 0.04, N = 4; two-way RM ANOVA Genotype × Phase p = 0.9901; Phase p = 0.0020; Genotype p = 0.4906), and (E) between CA1 and CA3 (WT vehicle: 0.40 ± 0.01, N = 5; MT 0.33 ± 0.01, N = 4; kainate: 0.359 ± 0.009, N = 5; MT 0.34 ± 0.01, N = 4; two-way RM ANOVA Genotype × Phase p = 0.9698; Phase p = 0.4348; Genotype p = 0.7785). F, Fold change in mean coherence from vehicle to kainate (Kruskal–Wallis statistic = 20.53; p = 0.001) within CA1 (WT: 1.01 ± 0.05, N = 5, MT: 1.18 ± 0.09, N = 4; p = 0.334), within CA3 (WT: 1.24 ± 0.12, N = 5, MT: 1.47 ± 0.08, N = 5; p > 0.999) and between CA1 and CA3 (WT: 0.908 ± 0.02, N = 5, MT: 1.04 ± 0.06, N = 4, p > 0.999). Data shown as mean ± SEM, N = number of slices. Kruskal–Wallis test and Dunn’s tests for multiple comparisons were performed in GraphPad Prism. Download Figure 7-4, TIF file. [file enu-eN-MNT-0167-23-s05.tif]
